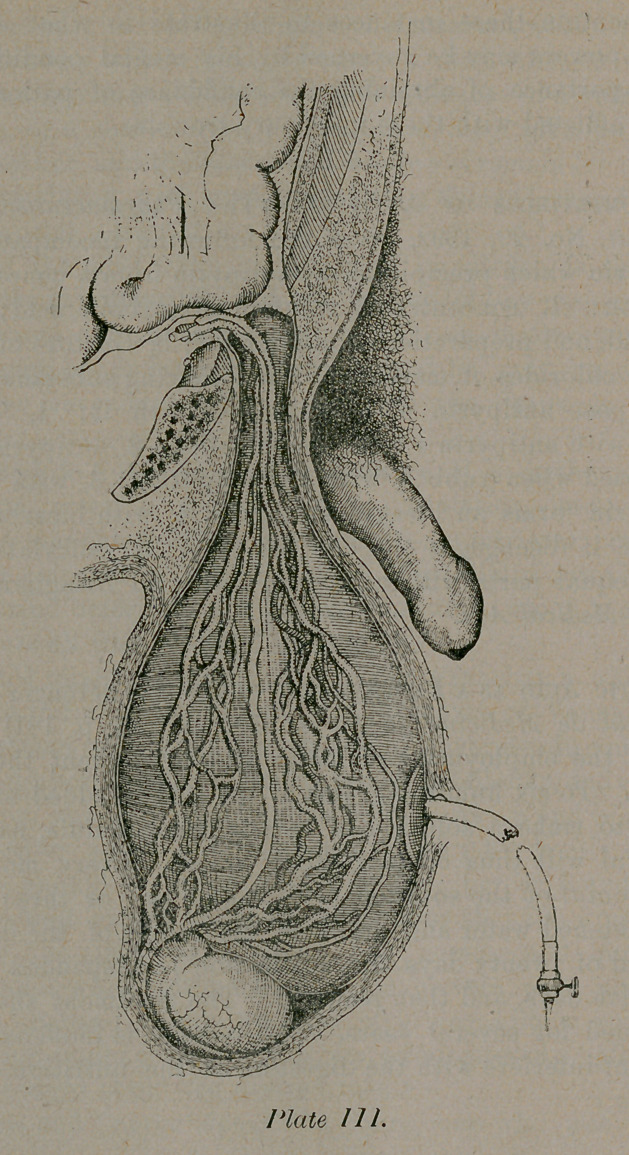# The Radical Cure of Hernia, Particularly with Children

**Published:** 1892-10

**Authors:** G. Felizet

**Affiliations:** Surgeon to Tenon Hospital, Paris, France


### THE RADICAL CURE OF HERNIA, PARTICULARLY WITH CHILDREN.

BY DR. G. FELIZET, SURGEON TO TENON HOSPITAL, PARIS, FRANCE.

Translated by Thos. H. Manley, A. M., M. D., Visiting Surgeon to Harlem Hospital, New York.

*(Continued.)*

### OPERATION FOR THE RADICAL CURE OF HERNIA.

Here many eventualities are to be considered.

(*A.*) *The hernia is entirely reducible.*

(*B.*) *The hernia is only partially reducible.*

(*C.*) *The hernia is absolutely reducible by adhesions.*

(*D.*) *Finally, after division of adhesions in a kelotomy for strangulated hernia, we may practice the operation at the same time for radical cure.*

(*a.*) The hernia is reducible in its totality.

This is the most simple variety and is the variety most common in children. It will serve us as a type. The easy return of the hernia is effected in the majority of cases.

But sometimes there are many loops of the intestine down, and their reduction, which *a priori* would seem a very simple matter, is very difficult and laborious, as we will sometimes find a fresh loop descend as the others are pressed backwards.

It was in a case of this description that I performed my second operation on a child 8 months old. The hernia was double, which unavoidably prolonged the operation beyond the limits to which we are accustomed.

The hernia being reduced, the finger is engaged in the opening of the sac which protects the two edges of the wound, and we explore things. This exploration must be rapid, but not too methodical. The finger is now carried to the base of the sac and the testis felt. If we touch the tunica albuginea, we are certain that we have what is anatomically known as a real congenital hernia. If, in an infant, we do not find the testis in the base of the scrotum, we must search for it. The finger is carried towards the ring. If the testicle is at the external orifice, it should be drawn downwards a little and engaged in the track, to assure not only the return of the intestine, but besides, to assure one that there are no adhesions involving the internal orifice. If the testis is at the internal orifice, the finger will test mobility and facilitate its descent. At last, if the testicle cannot be found; if it is perhaps recognized as an abdominal ectopy; that is to say, in such a condition in which it has escaped internal strangulation at the ring, it should be directed towards its normal passage.

I have always insisted that we should not hesitate because of these two latter eventualities. I have not yet met and operated for radical cure on these latter, as they are not numerous; so that in default of my own experience, I must have resort to that of other surgeons. If we should encounter this condition in an acquired or congenital hernia in an adult, with the testis having migrated into the abdomen, the important result of this exploration and operation would be to furnish him assurance against compression or incarceration of the organ of generation.

*3rd Stage*—With the finger which has explored the sac, the neck and abdomen, we quickly substitute the folded balloon; engaging its small extremity in the inguinal tract as deeply as possible. The balloon is now fixed in the sac; two sutures or forceps retract the opening above and below the tube, and in a minute it is insufflated. We see, while it is filling with air, the small extremity of the pear-shaped extremity of the air-bag engage in the inguinal tract, so that its apex is opposed to the retreating intestine during the operation. From this moment, the situation is simplified, and the procedure expedited.

We now have before us a hernia which we may deal with at our leisure; which we may attack with circumspection, free from all dangers; we have in reality, the ideal of a tumor, hard, glistening, regular, well circumscribed and mobile, which we may seize and enucleate with its peritoneal pedicle attached, which surrounds and distends it. Although the sac may have been primitively *cylindrical*, *conoid*, *globular*, or

*moniliform*, it is of no consequence, as it is now maintained of a regular pisiform shape. The shape of the sac has now no interest for us; what now interests us, is the neck of the sac, its exact dissection and its strict isolation up to the internal orifice. The entire rent in the sac being closed with the forceps, we make a circular section which presses the cellular envelopes until we see clearly the red tint of the gauze through the thin sac. We now introduce a grooved dissector, and with the bistoury or scissors we divide the coverings along the line of the cutaneous incision, as high as the external orifice of the inguinal canal. Guided by its pellucid hue, we are soon on the sac. The incision is continued downward in the same manner. From this, through the remainder, is but a matter of a few moments; with the scissors, finger and canular in, split the tunics, rendered clear by the uniform tint of our balloon. We are certain not to have left the contour of the protrusion, nor to have mutilated the soft parts, the preservation of which is necessary.

We are certain that we have preserved the elements of the cord; the spermatic canal, which is so small, so slightly visible in an infant; the spermatic veins, which we see in their sheaths, as the balloon rises, of a red and blue shade, without the loss of a single drop of blood. The spermatic artery is in great danger of being wounded, as it floats in its flexuosities in its bed of soft tissues, badly situated, obscure and but feebly supported. The nerves, one does not see, as they are blended with the other elements.

In some, the fingers meet posteriorly to the sac, and we may completely liberate the serous pellicle, after the manner in which we would enucleate a sarcocele. The greatest delay is in completely isolating the neck as high as possible. If, however, the incision into the integument is high enough and the rent in the distending bag is well maintained in the tract, the enterprise is easy. We must now make a certain traction on the peritoneum as low as possible, using the bistoury and tenaculum to freely separate it.

If the red tint of the rubber is everywhere fine and regular, we are sure the bottom only distends the sac, and that the intestine is not pressed or included. In order to be more positive yet, we execute a simple manœuvre. We give the sac two or three turns, while with the fine raspatory we scrape the pedicle till it is reduced to extreme thinness.

We may now proceed with every security, and transfix with a double suture the summit of the sac, using either catgut or silk, applying the knot tightly in the ordinary way. The section of the pedicle is made one centimetre below the divided pedicle. The end of the ligated sac quickly rises and is lost in the belly. We now protect the parts above with an antiseptic sponge. If the sac has been detached wholly below, we may now utilize what is left of it to facilitate its dissection.

We are now assured of a rapidity in operating without thoroughly freeing and seeing the sac and isolating its pedicle. Its density is a consideration, for with one hernia in an adult, measuring three or four times the size of the thumb, it weighed 3 grammes, and in one of children, recently operated on, 28 centigrammes. If a hernia appertains to the congenital variety, and if the testis is at the base of the sac, we must always preserve the peritoneum which constitutes its serous envelope. In this case it is easy, but it is not always necessary to suture. The serous surfaces are approximated, their borders trimmed sufficiently to assure space for the spermatic cord and tunica vaginalis.

With reference to sewing the pillars of the rings together, there is a question which demands further study, and hence, positive opinions must be reserved. What we wish to say is, that the operation of suturing them is very simple and harmless always, when we carry the incision of the integument far enough, dissecting in a systematic and methodical manner the sac from the diverging columns. Nothing will accrue from an operation which leaves the neck of the sac neither perfectly isolated, independent, nor continuous.

(*b.*) The hernia is reducible in part only.

We have so far considered only the least complicated. A simple sac, peritoneal or peritoneo-vaginal, free from all adhesions with the intestine or epiploon. These are the varieties most commonly met with in children. The epiploon which attaches itself with most facility to the parietal or visceral peritoneum, we may say, almost never forms a part of the contents of an infantile hernia, as when it does appear it must contract solid adhesions with the sac, an accident which seldom presents in early life.

Although such adhesions are exceptional, they are not impossible. When they do exist, they generally result from a truss which is badly adjusted, and excites inflammation. It may also be an incident of imperfect immigration of the testicle. The adhesions are less compact and solid than in an adult, sufficient time not having elapsed for fibrous changes to take place.

Though we may meet with the complication in an infant or child or adult, the distended balloon is equally efficacious in those cases, in the operation for their radical cure. The operation is commenced, as in the preceding cases which we have described, the surgeon proceeding cautiously until the neighborhood of the sac is reached. The difficulty now, is to know where we must make the opening. There is danger of cutting at once into an adherent intestine, if we do not alternately reduce and bring the intestine and pick up the sac, independent of its contents, when we may open it without fear.

Sometimes, it is extremely difficult to find the hernial investment. In one of our patients we had to divide successively many laminæ before we reached the hernial cavity. At last, the opening is made, the borders seized and drawn aside with the forceps. The finger enlarges the aperture and engages in the sac, when we recognize the seat, nature and extent of the adhesions.

In children, the adhesions will most commonly be found at the neck of the sac. If they are recent, the finger will easily separate them when reduction is easily affected. It must not be forgotten that the intestine at this early age is exceedingly fragile, and that we should accordingly manœuvre with extreme care. Certainly, if it does not lie in the way of the adhesions, there is nothing to fear.

With the adult, partial adhesions are observed everywhere; at the base of the sac, its sides, at the neck, and above all, in the abdominal funnel, near the internal orifice. The finger must thoroughly free them all. We now introduce the balloon through a slit in the sac, following its grand axis, engaging its small extremity in the inguinal tract, as close by as possible to the aperture. The balloon now filling and rising without bruising the parts contained, it carries them towards the surface. The surgeon now has under his eyes a tumor of a variable contour, but a consistence as usually encountered on which we practice a dissection and enucleation with as much facility, but a little more reserve, than in more happy cases. It is towards the free parts in the direction of the balloon that we first direct our dissection, its color directing us. It is on this we should depend for a guide and reliance, and then proceed with due care, freeing the adhesions and isolating the pedicle. Without any delay, we push the dissection towards the ring, in the meantime freeing the hernia from below.

There and there only, after having prepared by a dissection high up, we will attain a good result and radical cure. We now slowly allow the air to escape from the balloon, enlarge the slit in the sac superiorly, and fix the borders with forceps. The hernial contents are then examined and returned; the sac being fully and freely opened, we proceed to the liberations of the adhesions. If it is an adult, and the intestine is bound down by the omentum, it should be ligated and cut away; the intestine liberated, trimmed and returned. When the hernia is free, the finger is carried into the abdomen, to prove that all adhesions at the internal orifice are well loosened. A large forceps then seizes the neck and and draws the peritoneum as low down as we can, so that the catgut may be knotted as high as possible. With a child the introduction of the finger is necessary, as the neck with them is usually the seat of the obstacle. It is an adhesion due to a contusion by the truss, and is remedied without, in operating.

(*To be continued.*)

## Figures and Tables

**Plate III. f1:**